# An Enhanced TK Technology for Bearing Fault Detection Using Vibration Measurement

**DOI:** 10.3390/s25216571

**Published:** 2025-10-25

**Authors:** Megha Malusare, Manzar Mahmud, Wilson Wang

**Affiliations:** 1 Department of Mechanical and Mechatronics Engineering, Lakehead University, Thunder Bay, ON P7B 5E1, Canada; mmalusar@Lakeheadu.ca; 2 Electrical Department, Robotics and Space Operations, MTS Space, Brampton, ON L6Y 6K7, Canada; manzar.mahmud@mda.space

**Keywords:** fault detection, rolling element bearings, vibration measurement, Teager–Kaiser transform, signal processing

## Abstract

Rolling element bearings are commonly used in rotating machines. Bearing fault detection and diagnosis play a critical role in machine operations to recognize bearing faults at their early stage and prevent machine performance degradation, improve operation quality, and reduce maintenance costs. Although many fault detection techniques are proposed in the literature for bearing condition monitoring, reliable bearing fault detection remains a challenging task in this research and development field. This study proposes an enhanced Teager–Kaiser (eTK) technique for bearing fault detection and diagnosis. Vibration signals are used for analysis. The eTK technique is novel in two aspects: Firstly, an empirical mode decomposition analysis is suggested to recognize representative intrinsic mode functions (IMFs) with different frequency components. Secondly, an eTK denoising filter is proposed to improve the signal-to-noise ratio of the selected IMF features. The analytical signal spectrum analysis is conducted to identify representative features for bearing fault detection. The effectiveness of the proposed eTK technique is verified by experimental tests corresponding to different bearing conditions.

## 1. Introduction

Rolling element bearings are commonly used in rotating machines to support and facilitate shaft rotation and power transmission. A bearing is a mechanical system that consists of the outer ring, inner ring, rolling elements (balls or rollers), and a cage. As the bearing components are subjected to dynamic loadings in operation, they could be damaged for reasons such as fatigue and severe wear [1,2]. According to a previous investigation [3], more than 50% of rotatory machine imperfections are related to bearing faults. Therefore, new techniques of reliable bearing fault detection and diagnosis are critically needed in industries to recognize bearing defects as early as possible to prevent machine operation degradation, improve safety, and reduce costs of maintenance by preventing unnecessary machine downtime.

Fault detection is the process of applying some signal processing technique(s) to extract representative features from the measured signals to predict the health conditions of the machine, we use rolling element bearings in this work. Signals are measured by using appropriate sensors to transform physical quantities to electrical data. Bearing fault detection can be performed by analyzing signals in forms such as temperature, pressure, acoustics, lubricant, and vibration. Vibration signals usually have a higher signal-to-noise ratio (SNR) than other types of signals in machine fault detection [4], which will also be used in this work.

Bearing component materials are subjected to dynamic loading in operation. Bearing defects can occur when material fatigue limits are exceeded. Whenever the faulty location on a bearing element interacts with other bearing elements, abrupt changes in the contact stresses generate impact impulses, which will cause resonance vibrations of the bearing housing and its support structure. Based on the defect location, the respective characteristic frequencies for the bearing with an outer race defect ($f_{OD}$), inner race fault ($f_{ID}$), and rolling element damage ($f_{ED}$) are represented as [2]:

(1) $$f_{OD}=\frac{Z}{2}(1-\frac{d}{D}\cos(\theta ))\times f_{R}$$

(2) $$f_{ID}=\frac{Z}{2}(1+\frac{d}{D}\cos(\theta ))\times f_{R}$$

(3) $$f_{ED}=\frac{D}{d}(1-\frac{d^{2}}{D^{2}}\cos^{2}(\theta ))\times f_{R}$$

where *Z* is the number of rolling elements, $f_{R}$ is shaft rotating speed (Hz), *D* is the pitch diameter, θ is the contact angle, and *d* is the diameter of the rolling element.

Many techniques have been proposed in the literature for bearing fault detection. They can be classified into the time domain, frequency domain, and time–frequency domain analysis. In time domain analysis, bearing faults are detected by analyzing the vibration signal using some statistical indicators, such as skewness, root mean square, and kurtosis [5]. However, these methods have low accuracy in analyzing non-stationary signals, which usually correspond to faulty bearing features.

Signal processing can be performed in the frequency domain using techniques such as the Fourier Transform (FT) and cepstrum analysis. The spectrum of a vibration signal can be used to examine the theoretical characteristic frequencies for bearing fault detection [6]. However, the fault characteristic frequencies for many bearing fault conditions (e.g., inner race and rolling element faults) are non-stationary due to variations in load and rotational speed, slip in bearings, and nonlinear effects in the transmission system. Envelope analysis is another method in frequency domain analysis for bearing fault detection [7], which could overcome some of the shortcomings of the classical FT and the related methods. In envelope analysis, the signals are bandpass filtered, and only the signals around the resonance frequencies are applied to detect the bearing fault characteristic frequencies [8]. However, it is usually difficult to select proper frequency bands for envelope analysis.

Time–frequency domain analysis studies signal properties in both the time domain and the frequency domain simultaneously. There are several time–frequency signal processing methods in the literature, such as the wavelet transform (WT) [9] and the short-time FT [10]. WT analysis has low time resolution under high-frequency conditions and low-frequency resolution under high-time conditions, which ultimately undermines the accuracy of fault detection [11]. Moreover, if the signal changes due to an unexpected impact or noise, the original mother wavelet may not properly represent the signal properties in fault detection. To address these problems, empirical mode decomposition (EMD) has been applied for bearing fault detection. EMD is an adaptive data-processing method that provides multi-resolution over different frequency scales [12]. Using the instantaneous amplitude and instantaneous frequency, EMD can decompose the original signal into a set of intrinsic mode functions (IMFs). Several techniques have been proposed to select proper IMFs for bearing fault detection, for example, based on the energy associated with the IMF [13,14]. The fault representative IMF can also be selected based on correlation coefficients with the signal. However, EMD cannot decompose a signal strictly orthogonally. As a result, selecting one or two IMFs may lead to less reliable fault detection and sometimes make it difficult to apply EMD to long, non-stationary signals [15]. Hence, new IMF processing techniques are needed to address these limitations so as to provide more efficient and reliable bearing fault diagnosis.

In general, the Hilbert–Huang transform (HHT) performs better than the WT and short-time FT in bearing fault detection [13,16]. However, the HHT also has some limitations in edge distortion and mode mixing, which can degrade its processing accuracy [17]. The Teager–Kaiser (TK) energy operator needs only a few samples for energy calculation at each instant time instant for nonlinear and non-stationary signal processing. Several combined techniques have been suggested for bearing fault detection, for example, using a TK-energy transformation [1], TK-envelope technique [18], TK-energy operator [19], and TK-singular spectrum analysis [20]. Although TK analysis could provide better performance than the HHT in machine fault detection, the TK spectrums are very sensitive to high-frequency noise caused by speed variations, impacts, and sudden load changes.

To tackle the aforementioned limitations in existing techniques, the objective of this work is to propose an enhanced TK technique, eTK in short, for more accurate nonstationary signal analysis and bearing fault detection using vibration signals. The proposed eTK technique is new in the following aspects: (1) A new EMD analysis method is suggested to recognize the representative IMFs with different frequency components. (2) An eTK denoising filter is proposed to improve the signal-to-noise ratio (SNR) of the selected IMF features. The formulated analytical signal spectrum analysis is conducted to identify representative features for bearing fault detection. The effectiveness of the proposed eTK technique is verified through experimental tests conducted under different bearing conditions.

The remainder of the paper is organized as follows: The proposed eTK technique is discussed in Section 2. The effectiveness of the eTK technique is examined in Section 3 by systematic experimental tests.

## 2. The Proposed eTK Technique for Bearing Fault Detection

**Figure 1:**
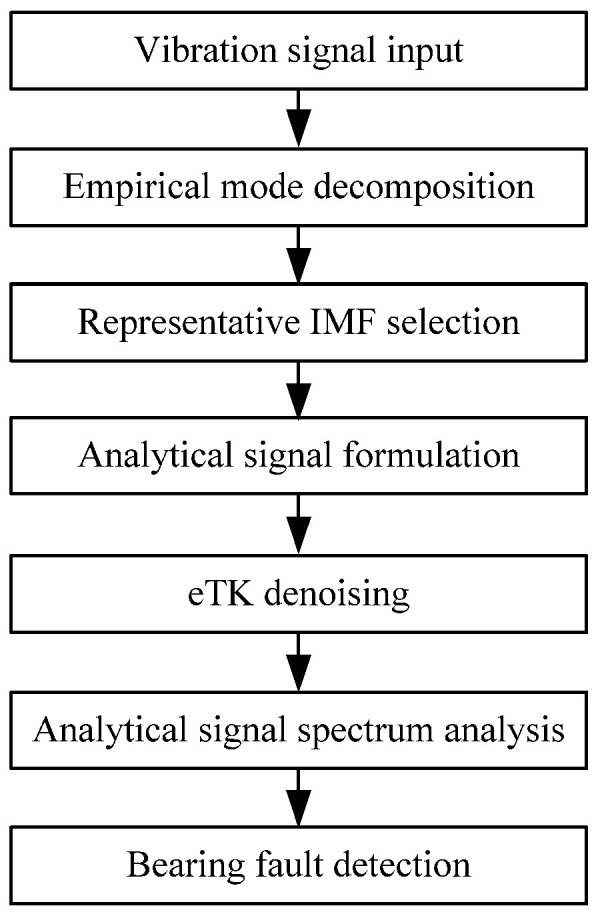
Flowchart of the eTK processing procedure.

Figure 1 shows the processing procedures of the proposed eTK technique; details will be discussed in this section.

### 2.1. Bearing Fault Analysis

Bearing fault typically begins with a tiny, localized defect, and then propagates with time to a severe stage. Bearing faults could generate excessive vibration and noise and lead to machinery malfunctions. Hence, it is highly important to recognize a bearing fault at its incipient stage. However, it remains a challenging task to reliably predict and diagnose bearing faults in machine condition monitoring because a rolling element bearing is a system, instead of a component. In addition, bearing representative features are often modulated due to the complex signal transmission path from the damaged location of the bearing to the sensors [2].

The common approach to detect bearing defects is to recognize the related fault characteristic frequencies as represented in Equations (1)–(3). When a bearing is damaged, whenever the damaged location strikes other bearing components, impacts are generated. The impacts then excite other bearing components, bearing housing, and support structures, and resonance vibrations are generated with the related fault frequencies. Due to the impedance effect of the transmission path from the faulty location to the sensors, the impact features are usually amplitude-modulated by other resonant frequencies of the bearings, and the impact feature magnitudes decay exponentially at the fault frequency [14]. In signal processing, this phenomenon is related to amplitude modulation (AM), which can be defined as a signal with its envelope varying with time such as,

(4) $$x_{AM}(t)=A_{m}(t)\cos(2\pi f_{c}t+\alpha )$$

where $A_{m}(t)$ represents the envelope function; $f_{c}$ and α are the frequency and phase of the carrier signal, respectively. For simplicity, the phase of the carrier signal can be assumed as zero. A DC offset can be added to the sinusoidal envelope function in Equation (4) to ensure that $A_{m}(t)$ is positive, or

(5) $$A_{m}(t)=1+b\cos(2\pi f_{m}t)$$

where *f_m_* is the modulating frequency, and *b* ∈ [0, 1] is the modulation index. The strength of the modulating signal depends on the value of *b*. Substituting Equation (5) in Equation (4) yields the following:

(6) $$x_{AM}(t)=\cos(2\pi f_{c}t)+b\cos(2\pi f_{m}t)\cos(2\pi f_{c}t)$$

Equation (6) can be further manipulated as,

(7) $$x_{AM}(t)=\cos(2\pi f_{c}t)+\frac{b}{2}\cos(2\pi (f_{c}-f_{m})t)+\cos(2\pi (f_{c}+f_{m})t)$$

**Figure 2:**
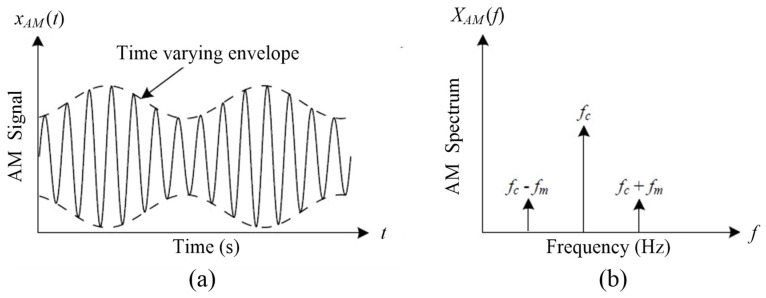
Example of an AM signal: (**a**) time domain signal, (**b**) frequency spectrum.

Thus, the AM signal $x_{AM}(t)$ can be represented as the summation of the carrier signal with frequency *f_c_* and the modulation sidebands (*f_c_* ± *f_m_*). Figure 2a illustrates an example of an AM signal with a time-varying envelope and the corresponding frequency spectrum with modulation sidebands. It is seen that the spectral components corresponding to the modulating frequency *f_m_* cannot be recognized directly on the spectral map in Figure 2b, but they become sidebands around the carrier frequency *f_c_*.

**Figure 3:**
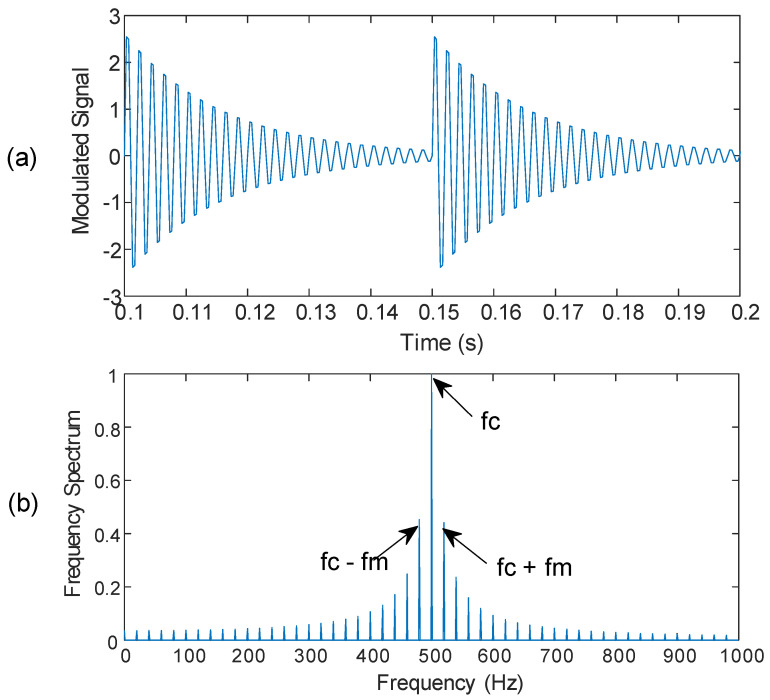
(**a**) Simulated resonance signature with modulation (a portion of the signal for illustration); (**b**) the corresponding frequency spectrum with modulation sidebands.

The resonance signature from a bearing can be assumed as a carrier signal in the AM. The amplitude of the carrier signal varies in proportion to the modulating signal. In the case of bearing fault, the amplitude of the resonance signature (i.e., carrier signal) will change due to the impact (i.e., modulating signal). Figure 3 shows a simulated resonance signal and the corresponding frequency spectrum with modulation sidebands.

### 2.2. The Proposed eTK Denoising Filter

The proposed eTK denoising filter aims to reduce the noise and enhance the SNR. The energy of a signal is usually represented by the square of the amplitude, or

(8) $$E(t)=|x(t){|}^{2}$$

However, the energy in a signal also depends on higher frequency signatures. Hence, instantaneous energy analysis will be applied in this work for signal processing. The TK is a nonlinear differential operator that can track the instantaneous energy of the signal [1]. For a continuous signal *x*(*t*), the TK operator, ψ, is defined as,

(9) $$\psi (x(t))=\dot{x}t^{2}-x(t)\ddot{x}(t)$$

where $\dot{x}(t)$ and $\ddot{x}(t)$ represent the velocity and acceleration of the collected vibration signal x(t), respectively. For a discrete signal *x*[*n*], the TK can be calculated by,

(10) $$\psi \left[x[n]\right]=x^{2}[n]-x[n+1]\;x[n-1]$$

It is seen from Equation (10) that only three consecutive samples are needed to calculate the instantaneous energy in the signal. Therefore, TK can characterize the instantaneous changes in the signal to resolve transient events. However, the traditional TK operator in Equation (10) is very sensitive to high-frequency noise such as spikes and sudden changes [4]. The noise sensitivity over higher frequency bandwidth can be reduced by,

(11) $$\psi \left[x[n]\right]=x^{2}[n]-x[n+K_{i}]\;x[n-K_{i}]$$

where $K_{i}$ is the initial lag parameter, representing the distance between the samples. The optimal lag *K* can be estimated by,

(12) $$K=\frac{f_{s}}{2f_{m}},\;\mathrm{if}\;f_{m}\leq f_{s}$$

(13) $$K=\frac{f_{s}}{f_{m}},\;\mathrm{if}\;f_{m}>f_{s}$$

where *f_c_* is the carrier frequency and *f_m_* is the modulating frequency.

The energy in Equation (11) has a squared term to incorporate instantaneous frequency, which can change the scale of the signal and may make the results difficult to describe. In the proposed eTK, an indicator is introduced to describe standard deviations of instantaneous energy, which is defined as,

(14) $$Z_{TK}=\frac{T_{K}-{\bar{T}}_{K}}{\sqrt{\frac{1}{N}\sum_{i=1}^{N}{\left(E_{i}-\bar{E}\right)}^{2}}}$$

where $E_{i}$ and $\bar{E}$ represent the respective instantaneous energy and mean energy of the signal; *N* is the total number of data points in the signal; and $\bar{T}$ represents the mean of TK.

**Figure 4:**
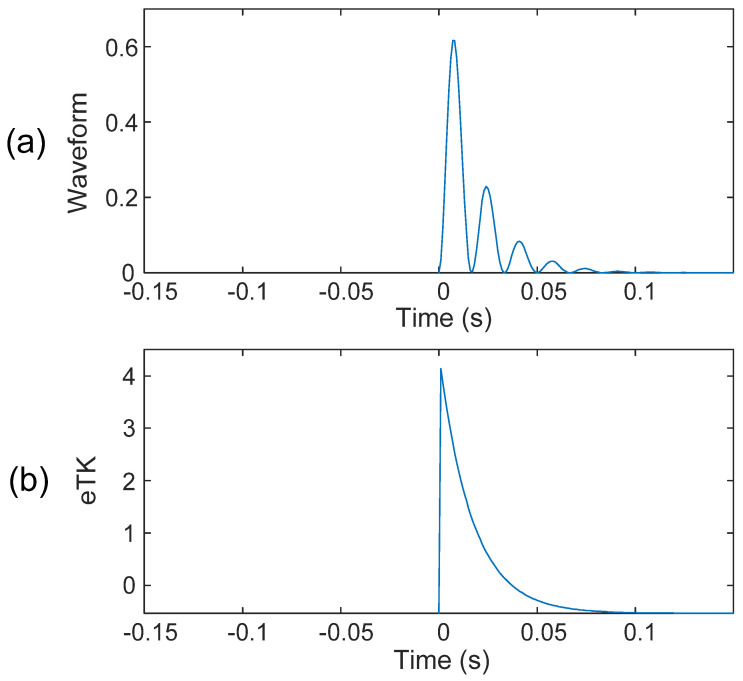
(**a**) Simulated resonance waveform. (**b**) The results after denoising using the eTK technique.

The eTK method takes the standard deviation of the instantaneous energy, which does not change the scale of the signal, to facilitate the analysis. Figure 4a shows a simulated waveform containing some side spikes along the main spike. As illustrated in Figure 4b, the eTK can clearly denoise the signal, smoothen the side spikes, and increase the SNR.

The primary factors determining the attenuation and oscillation period in the vibration signal are the resonance frequencies of the system, which are related to the signal transmission impedance, physical stiffness of the bearing–housing structure, and damping effects, etc. In the context of the eTK denoising filter, the most important factor controlling the output is the lag parameter *K_i_*. A larger value of *K_i_* increases spacing and enhances the low-frequency response, which produces longer oscillatory periods and smoother transient attenuation. In contrast, a smaller value of *K_i_* produces a high frequency response that resolves sharper impulses, but the response could be noise affected. Therefore, the proper selection of *K_i_* can balance the sensitivity to impulses versus noise elimination in eTK filtering.

### 2.3. The Proposed IMF Selection Method in the eTK

**Figure 5:**
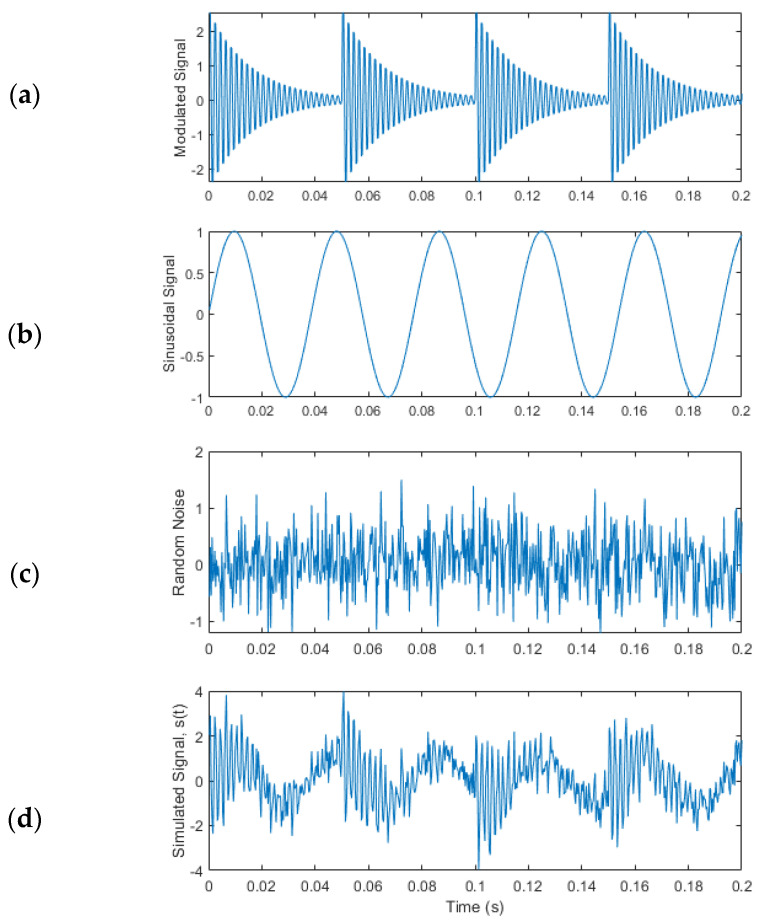
Simulated AM signal: (**a**) modulated signal, (**b**) sinusoidal signal, (**c**) random noise, and (**d**) final simulated signal *s*(*t*).

The EMD is an adaptive signal processing method that can be utilized for nonlinear and non-stationary signal processing [21]. To implement EMD, a simulated AM signal is created. The simulated AM signal is simulated, which consists of a modulated signal (Figure 5a), a sinusoidal signal (Figure 5b), and some random noise (Figure 5c) with a magnitude of about 45% of the modulated signal amplitude. Figure 5d shows the final simulated AM signal *s*(*t*).

The EMD decomposes a signal into a finite set of IMFs. Each IMF component must satisfy the criteria: (1) the difference between the number of extrema and the number of zero crossings must be zero or one; and (2) the mean value of the envelope from the local minima and local maxima must be zero. The IMFs are determined based on the following iterative sifting processes:

(1) If s(t) is the input signal, its upper envelope $s_{U}(t)$ and lower envelope $s_{L}(t)$ are computed by interpolation (e.g., the cubic spline function) where the set of control points are the local maxima and local minima of the signal.

(2) The mean value of the upper and lower envelopes will be

(15) $$M_{11}(t)=\frac{s_{U}(t)+s_{L}(t)}{2}$$

(3) The difference between the upper envelope and the mean value is,

(16) $$H_{1}(t)=s_{U}(t)-M_{11}(t)$$

(4) If *H*_1_(*t*) satisfies both the conditions of an IMF, then it is considered as the first *IMF* or *IMF*_1_. Otherwise, *s*(*t*) is replaced by *H*_1_(*t*) as the new primary signal. Steps (1) to (3) are repeated to find the first IMF:

(17) $$H_{11}(t)=H_{1}(t)-M_{11}(t)$$

The sifting processes in Equation (17) are repeated over *k* times, until *H*_1*k*_(*t*) becomes an IMF denoted as,

(18) $$C_{1}(t):=H_{1k}(t)$$

(5) The residual signal is calculated as,

(19) $$R_{1}(t)=s(t)-C_{1}(t)$$

(6) All possible IMFs inherited to signal *s*(*t*) are calculated by repeating the processes in Equations (15)–(19):

(20) $$R_{n}(t)=R_{n-1}(t)-C_{n}(t),\;n\;=\;2,3,\ldots ,\;N$$

**Figure 6:**
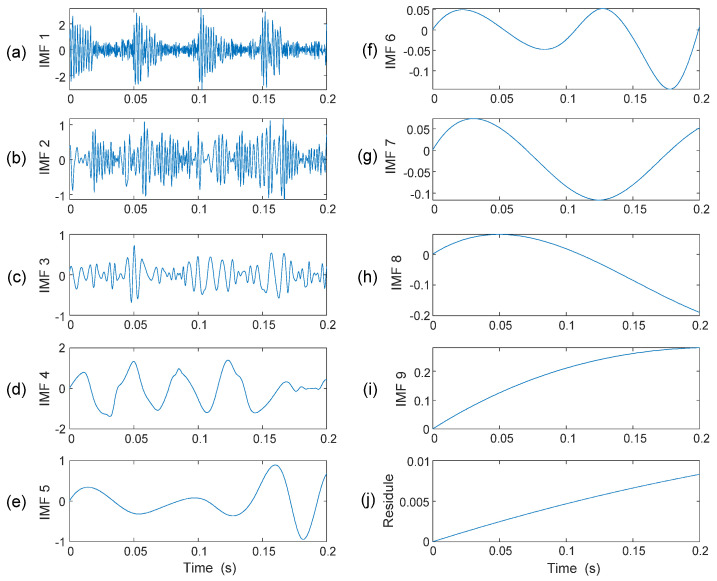
(**a**–**i**) IMFs formulated on signal *s*(*t*); (**j**) the residual signal.

The decomposition process is terminated when *R_n_*(*t*) becomes a mono-component function. If the above IMF shifting procedures are applied on the simulated signal *s*(*t*), as shown in Figure 5d, the resulting IMFs are calculated and are illustrated in Figure 6.

Since the signal energy is contained mainly in the first few IMFs, the classical approach is to take the first one or two IMFs for analysis [16]. However, it may not be always the case in bearing fault detection as the bearing is a system with complex vibration signal properties. A general approach to select representative IMFs is to use a correlation coefficient [21]. However, it is observed that the dot product between two IMFs may not be zero, or EMD cannot strictly decompose IMFs orthogonally [22]. As a result, it is difficult to predict if the first IMF always covers the fault-related features. To tackle this problem, a new IMF selection method is proposed in this work to select the most representative IMFs.

Consider the example in Figure 6. If it is decomposed into nine IMFs, IMF_1_~IMF_9_, and a residual, it is seen that only the first three IMFs in Figure 6a–c contains a specific distortion pattern, while other IMFs do not contain a clear, specific distortion pattern (Figure 6d–j). The feature in Figure 6c is not completely periodic, but quasi-periodic, because the extracted IMFs are modulated by other resonance responses and noise. In a bearing signal, for example, features could be quasi-periodic because they are modulated by shaft rotation variations, slips, and load changes.

**Figure 7:**
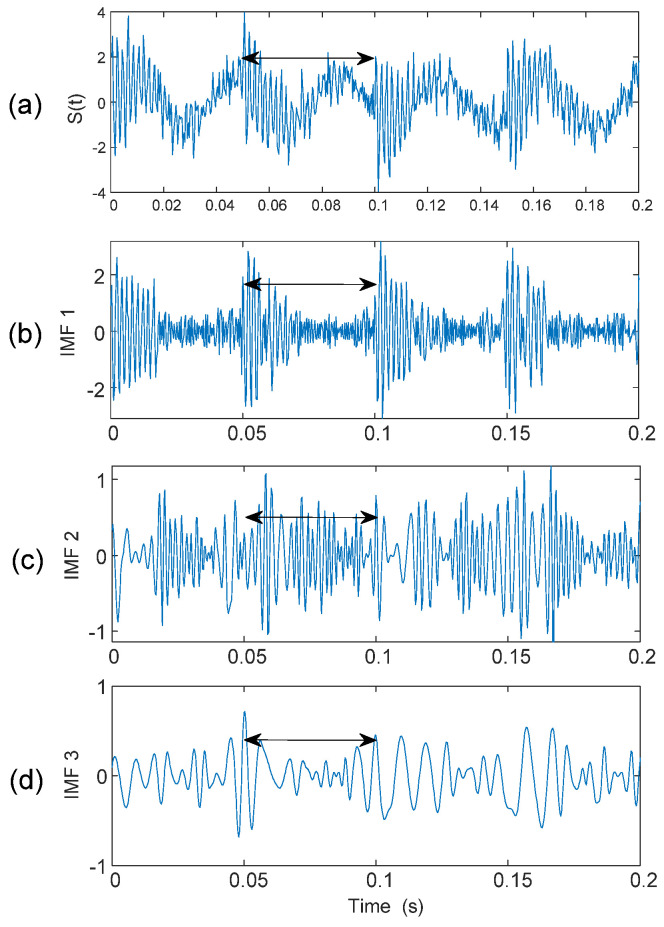
Distortion patterns (shown by a double-sided arrow) present in: (**a**) the simulated signal, (**b**) the first IMF, (**c**) the second IMF, and (**d**) the third IMF.

When a bearing is damaged, the resulting impacts will modulate the corresponding characteristic features. From our previous tests and investigations [2,15], these oscillatory components could correspond to bearing fault-related features. Hence, the first three IMFs will be selected for advanced analysis in this case. In real-time bearing fault detection, the specific distortion pattern should match with the corresponding critical fault frequencies. Figure 7 illustrates the distortion pattern presented in the simulated signal *s*(*t*), and in the first three IMFs.

### 2.4. The eTK Technique for Bearing Fault Detection

As discussed in Section 2.3, the most representative IMF patterns will be selected for analysis. These selected IMFs are then synchronized to form the analytical signal. In processing, each time, *t*, can be represented by its nearest distinct point, *d*, and each *IMF_k_*(*t*) is transformed into a distinct point representation, *IMF_k_*(*d*). Or the local band, *B_w_*, can be transformed into *D_w_* by

(21) $$D_{w}=2R\;\langle \frac{1}{2}B_{w}\frac{D_{s}}{F_{s}}\rangle$$

where $F_{s}$ is the sampling frequency in Hz, $D_{s}$ is the discrete representation of $F_{s}$, and $R\langle •\rangle \;$ represents the round-off operation.

Then, the representative features in the *k*th IMF can be transformed from *t_F_*(*k*) to the nearest distinct point *d_F_*(*k*), where *k =* 1, 2,…, *K*, and *K* is total number of IMFs selected to formulate the analytical signal. The *k*th local band, $\lambda_{k}$, in the distinct transform can be determined by

(22) $$\lambda_{k}=\left\{IMF_{k}(u)\right\};\;u=d_{1}(k),\;d_{2}(k),\;\ldots ,\;d_{F}(k),\;\ldots ,\;D_{w}$$

where distinct points *d*_1_(*k*), *d*_2_(*k*), …, *d_F_*(*k*), …, *D_w_* at the *k*th local band correspond to *t*_1_(*k*), *t*_2_(*k*), …, *t_F_*(*k*), …, *B_w_* of the *k*th IMF, respectively.

The *u*th distinct point in the *k*th local band $\lambda_{k}$, denoted as $\lambda_{u,\;k}$, will be sorted in an ascending order. The formulated analytical signal becomes

(23) $$S_{A}=\sum_{k=1}^{K}\lambda_{u,k};\;u=d_{1}(k),\;d_{2}(k),\;\ldots ,\;d_{F}(k),\;\ldots ,\;D_{w}.$$

**Figure 8:**
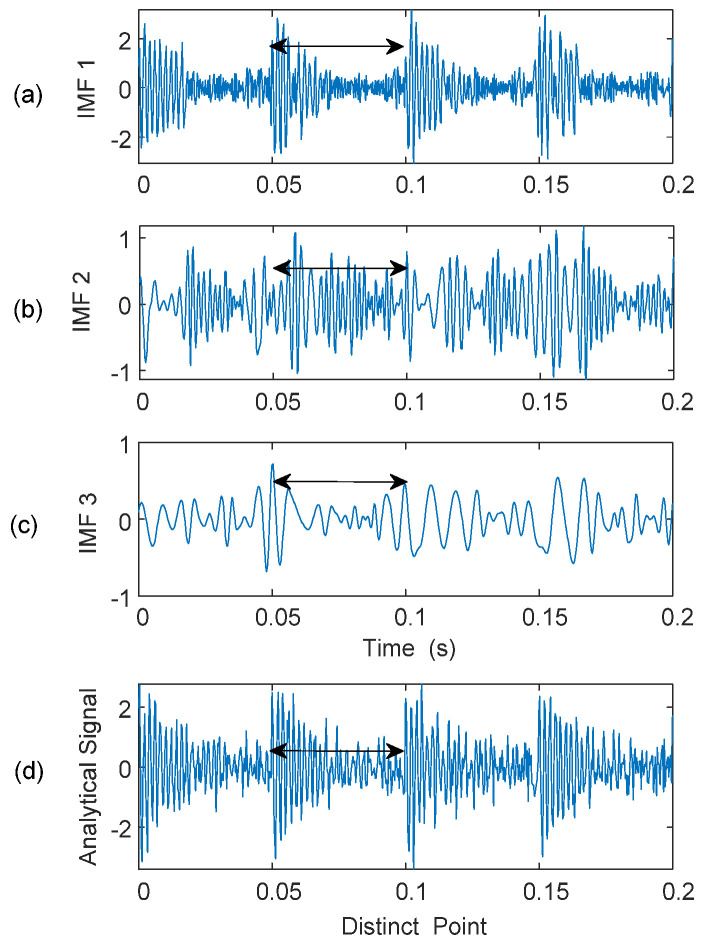
Formulation of the analytical signal from first three IMFs. Distortion patterns are indicated by double-sided arrows. (**a**) IMF 1 showing high-frequency impulsive transients; (**b**) IMF 2 exhibiting mixed modulation components; (**c**) IMF 3 dominated by the low-frequency resonance component; (**d**) Formulated analytical signal obtained by combining the selected IMFs.

Figure 8 illustrates the formulated analytical signal, $S_{A}$, from IMF_1_, IMF_2_, and IMF_3_. Based on the formulated analytical signal, the proposed eTK technique will be applied for signal property analysis and bearing fault detection. The eTK method is used to detect carrier signatures from modulated sidebands.

**Figure 9:**
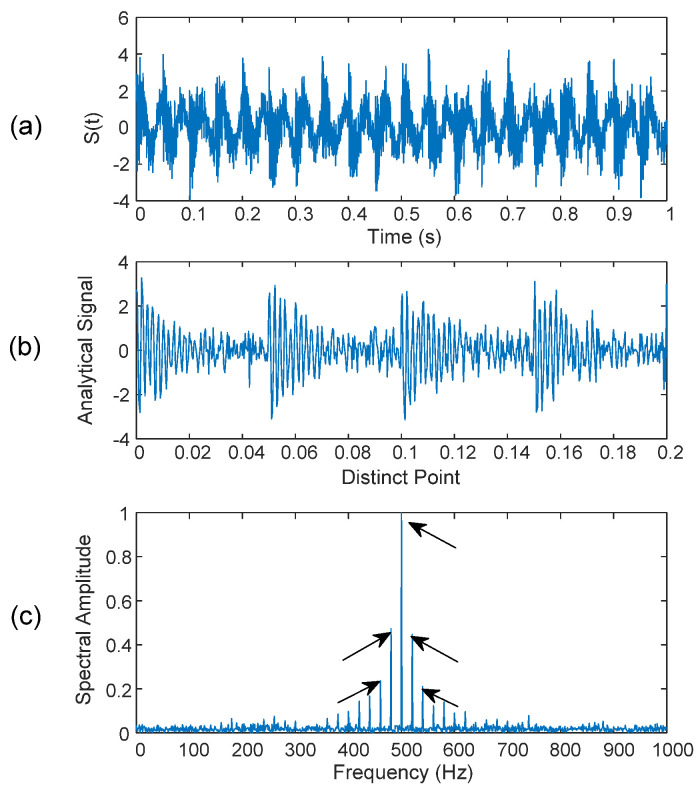
(**a**) Modulated signal *s*(*t*), (**b**) analytical signal, and (**c**) spectrum of the analytical signal. The arrows in (**c**) indicate the carrier frequency and the corresponding modulation sidebands of the analytical signal.

Figure 9 shows the modulated AM signal, the formulated analytical signal, and the spectral spectrum of the analytical signal. It is seen from Figure 9c that the carrier frequency and modulated sidebands can be recognized clearly (specified by arrows). The noise level due to unwanted frequencies is almost negligible in the spectral map. This is because the analytical signal is formulated only from those most representative IMFs that show critical distortion patterns, without considering other noise-related feature distortions. In this example, the simulated AM signal has the first three sequential IMFs containing specific distortion patterns. However, in real-time bearing fault detection, defect-induced distortion may not be present in sequential IMFs, which depend on the bearing’s dynamics and system structures, as illustrated in the tests in the next section.

## 3. Performance Verification

The effectiveness of the proposed eTK technique will be examined in this section by experimental tests using vibration signals. Its robustness will be tested using datasets from a different experimental setup.

### 3.1. Experimental Setup

**Figure 10:**
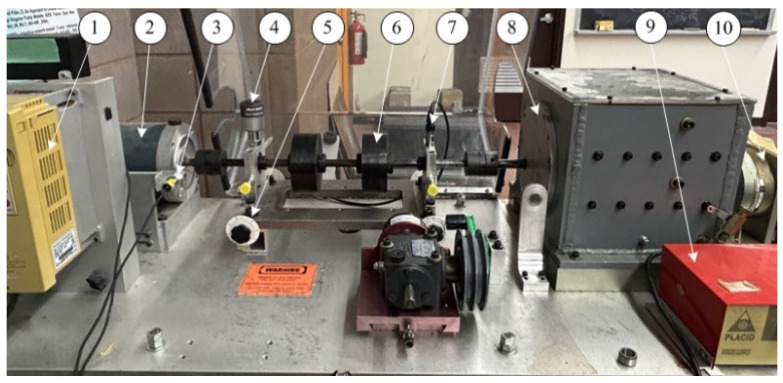
Experimental setup: (**1**) speed control; (**2**) drive motor; (**3**) optical encoder; (**4**) a smart sensor node; (**5**) misalignment adjustor; (**6**) static load; (**7**) ICP accelerometer; (**8**) gearbox; (**9**) load drive system; and (**10**) load system.

Figure 10 shows the experimental setup used in this test. It is driven by a 3 HP electric motor operating at speeds ranging from 100 to 4200 rpm, regulated by a frequency converter (VFD022B21A, WiAutomation, CA, USA). Elastic couplings are utilized to eliminate high-frequency impacts and vibrations from the motor and the gearbox. An optical sensor provides a one-pulse-per-revolution signal to measure shaft speed. The bearing under test (MBER-10K, MAT, ON, Canada) is located on the left bearing housing, with the following bearing parameters: eight balls, ball diameter of 7.938 mm, a pitch diameter of 33.503 mm, and a contact angle of 0°. Static loads are applied using two heavy mass disks and a dynamic load is introduced through a brake system connected via a gearbox. Vibration signals are acquired using smart vibration sensors developed by the authors’ research team. General accelerometers (ICP-603C01) mounted on the top of another bearing housing are used for verification. The collected signals are processed and analyzed using MATLAB R2024a (MathWorks, Natick, MA, USA).

In this test, four bearing health conditions are considered: healthy bearings, bearings with outer race defects, bearings with inner race defects, and bearings with rolling element defects. Table 1 summarizes the characteristic frequencies in terms of shaft speed $f_{R}$ for bearings with different health conditions using Equations (1)–(3).

### 3.2. Test Result Analysis

The performance of the proposed eTK technique includes denoising and IMF synthesis. It is represented as eTK, which is compared with two other related techniques.

(1) To compare the effectiveness of the proposed eTK, another related technique named HHT is used for comparison, specified as HHT.

(2) To verify the necessity of the denoising process, a comparison is provided with the proposed eTK but without using the denoising filter, denoted as TK.

All the techniques are implemented in MATLAB R2023b. Many tests have been undertaken under different speed and load conditions. A set of typical processing results with shaft speed of *f_R_* = 30 Hz (or 1800 RPM), load level of 2.3 Nm, and sampling frequency of 20 kHz, are used for illustration.

To quantify the fault detection effectiveness using the related techniques, a diagnostic clarity index is adopted for evaluation:

(24) $$D_{I}=\frac{\sum_{f\in f_{c}}\left(S(f)/S_{\max}(f)\right)}{\sum_{f}\left(S(f)/S_{\max}(f)\right)}$$

where *S*(*f*) is the spectral amplitude at frequency *f*, which is normalized by the maximum spectral amplitude $S_{\max}(f)$ over the bandwidth; *f_c_* denotes a bearing characteristic frequency and its first three harmonics.

#### 3.2.1. Healthy Bearing Analysis

**Figure 11:**
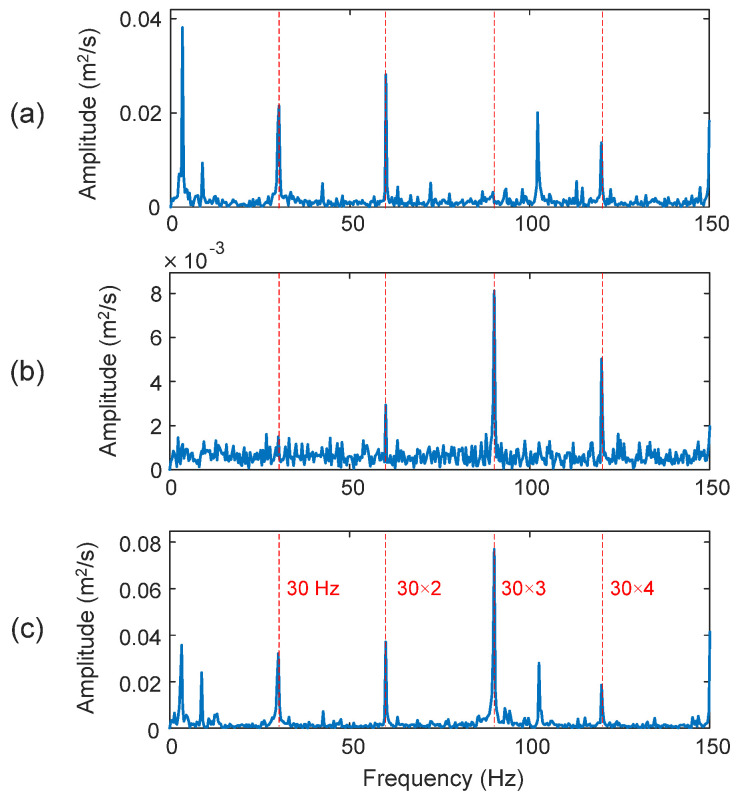
Processing results of a healthy bearing using the techniques of: (**a**) HHT, (**b**) TK, and (**c**) eTK. The red dashed lines represent the characteristic frequency *f_H_* = 30 Hz and its harmonics.

Firstly, the tests are undertaken on a healthy bearing. The bearing characteristic frequency in this case is *f_H_* = 30 Hz. Figure 11 shows the processing results using related techniques. The selected IMFs are the first and second IMFs (i.e., IMF_1_ and IMF_2_). As shown in Figure 11a, the classical HHT can recognize the characteristic frequency (*f_H_* = 30 Hz) and its harmonics; however, it does not dominate the resulting spectrum, which may result in false diagnosis; its diagnostic clarify index is *D_I_* = 87.5%. Examining the TK in Figure 11b, it is seen that TK, or eTK without denoising, can only recognize the third harmonic of the characteristic frequency with a very low magnitude (*D_I_* = 90.8%). On the other hand, the eTK with the denoising filter performs better than the TK method, which not only has a much higher magnitude (0.08 vs. 0.008), but also can recognize the fundamental characteristic frequency (*f_H_* = 30 Hz) clearly, as shown in Figure 11c with *D_I_* = 96.5%.

#### 3.2.2. Outer Race Fault Detection

**Figure 12:**
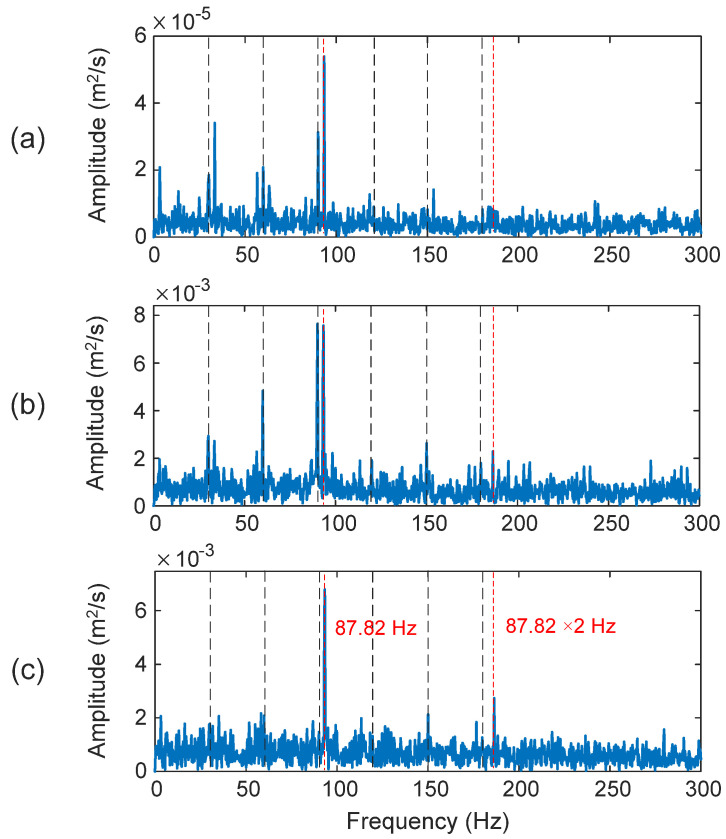
Processing results of a bearing with an outer race fault using the techniques of: (**a**) HHT, (**b**) TK, and (**c**) eTK. The red dashed lines represent fault characteristic frequency (87.82 Hz) and the black dashed lines represent shaft frequency (30 Hz) and its harmonics.

The outer race is the fixed ring in most bearing applications. When a bearing is damaged, the amplitude modulation of fault characteristic frequency is usually masked by strong noise. The selected IMFs in this case are the first, second, and fourth IMFs (i.e., IMF_1_, IMF_2_, and IMF_4_). Figure 12 shows the processing results using related techniques. In this case, the characteristic fault frequency is *f_OD_* = 87.82 Hz. As shown in Figure 12b, although the KT can recognize the fault characteristic frequency, it is very close to the third harmonic of the shaft speed, but with a lower magnitude. Correspondingly, the diagnostic information is not clear, with a *D_I_* = 79.6%. Examining Figure 12a, the HHT method can clearly predict the bearing outer race fault with *D_I_* = 95.3%. However, in comparison with the processing results of the proposed eTK technique in Figure 12c, it is seen that the eTK method performs better, which exhibits higher spectral magnitude at the fault frequency and its second harmonic (*D_I_* = 99.4%) because of its unique feature selection and denoising effects.

#### 3.2.3. Inner Race Fault Detection

**Figure 13:**
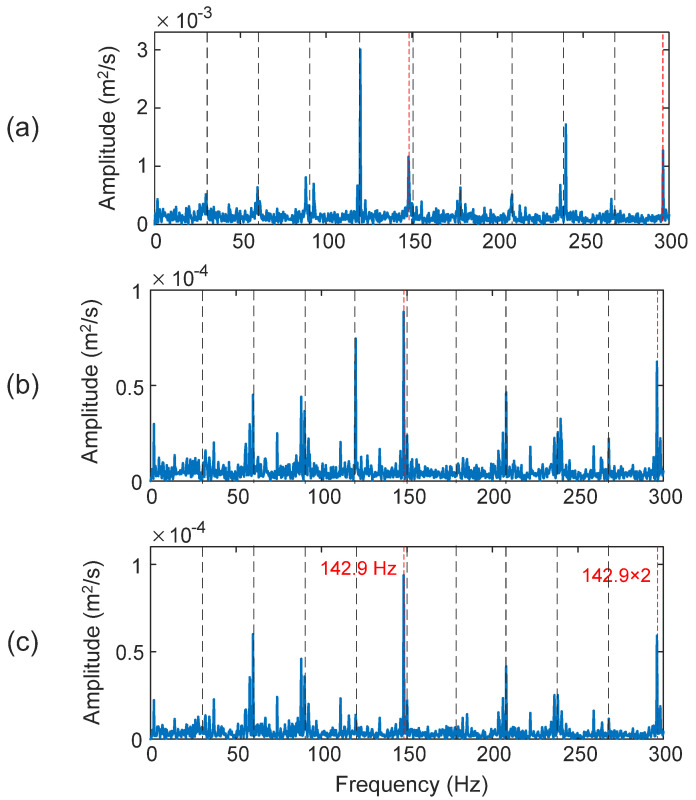
Processing results of a bearing with an inner race fault using the techniques of: (**a**) HHT, (**b**) TK, and (**c**) eTK. The red dashed lines represent fault characteristic frequency (142.9 Hz) and the black dashed lines represent shaft frequency (30 Hz) and its harmonics.

The second and third IMFs (i.e., IMF_2_ and IMF_3_) are selected for inner race fault detection. The fault characteristic frequency in this case is *f_ID_* = 142.9 Hz. As shown in Figure 13a, although the HHT can recognize the fault characteristic frequency and its harmonic in this case (*D_I_* = 72.3%), the shaft rotating frequencies dominate the spectrum, which degrades fault detection reliability. The inner race rotates with the shaft, which makes it difficult to detect the fault spectral features, especially considering the slip and load zone dynamic variations. Both the TK method in Figure 13b and the eTK technique in Figure 13b clearly predict the occurrence of the bearing inner race defect, in this case with dominant fault characteristic frequency, the eTK in Figure 13b performs even better than the TK, because the eTK denoising filter can effectively improve the SNR and highlight the fault features against noise. In this case, the diagnostic clarity index of TK is *D_I_* = 97.3% and the eTK technique is *D_I_* = 99.5%.

#### 3.2.4. Rolling Element Fault Detection

**Figure 14:**
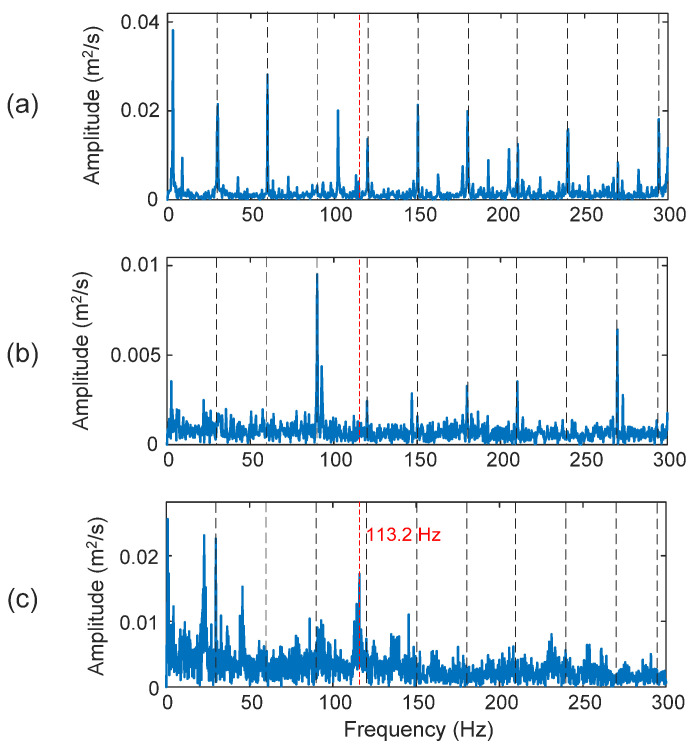
Processing results of a bearing with a rolling element fault using the techniques of: (**a**) HHT, (**b**) TK, and (**c**) eTK. The red dashed lines represent fault characteristic frequency (113.2 Hz) and the black dashed lines represent shaft frequency (30 Hz) and its harmonics.

Detecting faults in a rolling element (a ball) is usually the most challenging task in bearing fault detection. In this case, the fault characteristic frequency is *f_BD_* = 113.2 Hz. The selected IMFs are the first, second, and third IMFs (i.e., IMF_1_, IMF_2_, and IMF_3_). As shown in Figure 14a,b, both the HHT and TK have failed to recognize the fault features to predict the bearing fault in this case, with *D_I_* = 12.4% and 7.6% for the HHT and TK, respectively. The proposed eTK, however, is the only technique that can provide some indication of the rolling element damage, as shown in Figure 14c, even though the feature does not dominate the spectrum (*D_I_* = 77.8%). It is because a ball rotates as well as slides, which makes the fault resonance modes change over time. The vibration patterns change when the damaged ball moves from the load zone to the unload zone. Complex impacts and vibrations are generated due to these effects.

### 3.3. Robustness Testing

**Figure 15:**
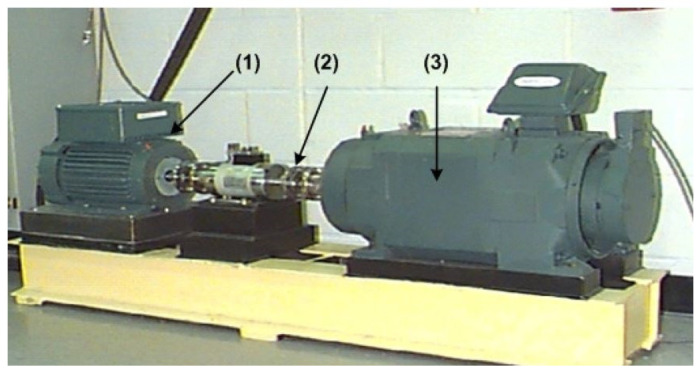
CWRU experimental setup [23]: (1) drive motor, (2) tested bearing, and (3) load motor.

To evaluate the robustness of the proposed eTK technique, different vibration datasets from Case Western Reserve University (CWRU) [23] are used for this investigation. Experiments are conducted using the experimental setup, as shown in Figure 15. The system is driven by a 2 hp motor. The vibration signals are measured using accelerometers attached to the housing using magnetic bases. Accelerometers are placed at both the drive-end and fan-end of the motor housing. Data from the drive-end is used for analysis in this work. Vibration signals are collected using a sampling frequency of 12,000 Hz. More details about the bearing test conditions can be found in [23].

The tested bearings are 6205-2RS JEM, SKF, CA, USA (deep groove ball bearing from SKF), with the following parameters: rolling elements, *Z*: 8; rolling element diameter, *d*: 7.94 mm; pitch diameter, *D*: 39.04 mm; and contact angle, θ: 0 degree.

The tested bearings have different health conditions (e.g., healthy, outer, inner, and rolling element faults). By Equations (1)–(3), the corresponding characteristic frequencies in terms of shaft rotation speed $f_{R}$ are summarized in Table 2.

#### 3.3.1. Healthy Bearing Condition Monitoring (Simulation Test)

**Figure 16:**
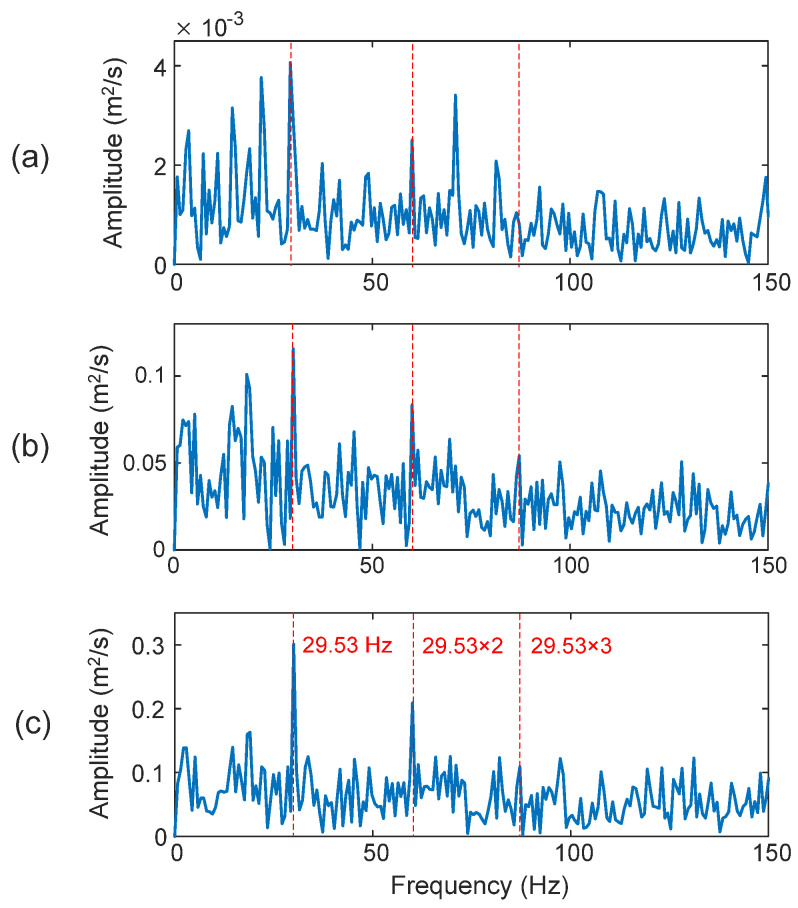
Processing results of healthy bearings using the techniques of: (**a**) HHT, (**b**) TK, and (**c**) eTK. The red dotted lines represent characteristic frequency (29.53 Hz) and its harmonics.

Firstly, processing results of a healthy bearing using the same techniques are shown in Figure 16. In this case, the characteristic frequency of the bearing is *f_H_* = 29.53 Hz. It is seen that the TK in Figure 16b and eTK in Figure 16c perform better than the HHT (*D_I_* = 87.6%) in Figure 16a. On the other hand, the eTK (*D_I_* = 98.7%) outperforms TK (*D_I_* = 90.5%), because the eTK has a higher SNR using its efficient denoising filter.

#### 3.3.2. Outer Race Fault Detection

**Figure 17:**
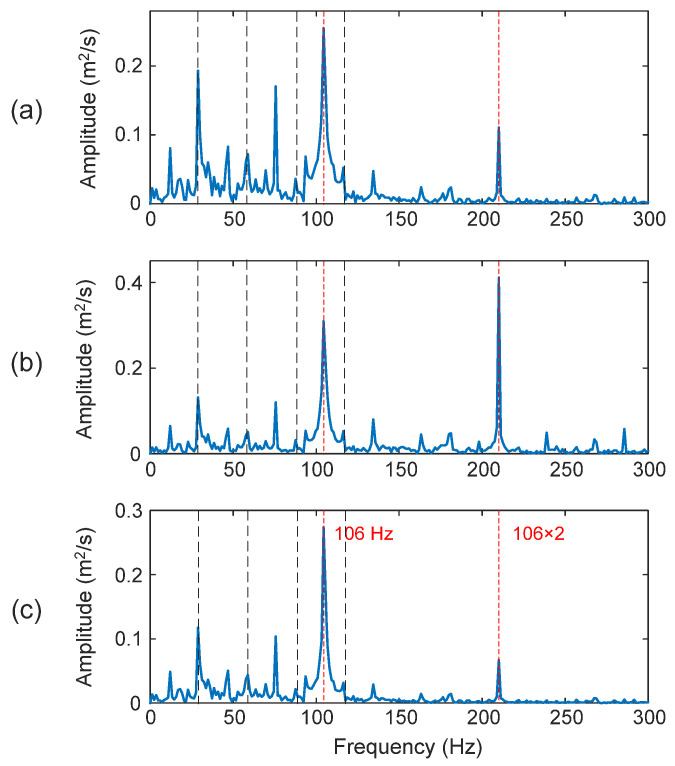
Processing results of a bearing with outer race fault using the techniques of: (**a**) HHT, (**b**) TK, and (**c**) eTK. The red dashed lines represent fault characteristic frequency (106 Hz) and the black dashed lines represent shaft frequency (29.53 Hz) and its harmonics.

Figure 17 shows the processing results for a bearing with an outer race defect. In this case, the fault characteristic frequency is *f_OD_* = 106 Hz. Although all three techniques can predict the outer race bearing fault, the proposed eTK technique in Figure 17c performs the best with *D_I_* = 99.1%, due to its efficient denoising filtering improve the SNR. The HHT method in Figure 17a has a higher noise level (*D_I_* = 92.2%) than the eTK. Even though the TK in Figure 17b can predict the bearing fault, the dominant frequency is the second harmonic of the characteristic frequency (*D_I_* = 90.5%).

#### 3.3.3. Inner Race Fault Detection

**Figure 18:**
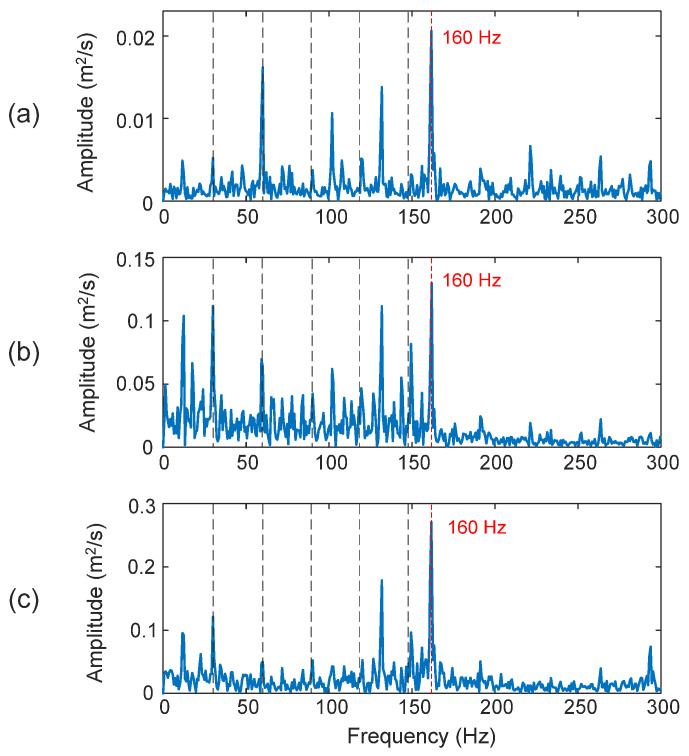
Comparison of processing results of a bearing with an inner race fault using the techniques of: (**a**) HHT, (**b**) TK, and (**c**) eTK. The red dashed lines represent fault characteristic frequency (160 Hz) and the black dashed lines represent shaft frequency (29.53 Hz) and its harmonics.

The processing results of a bearing with an inner race fault are shown in Figure 18. The fault characteristic frequency in this case is *f_ID_* = 160 Hz. It is seen that the proposed eTK technique (*D_I_* = 98.7%) in Figure 18c outperforms the HHT (*D_I_* = 91.4%) in Figure 18a and the TK method (*D_I_* = 88.5%) in Figure 18b, in terms of SNR and clarity in diagnosing fault characteristic frequency.

#### 3.3.4. Rolling Element Fault Detection

**Figure 19:**
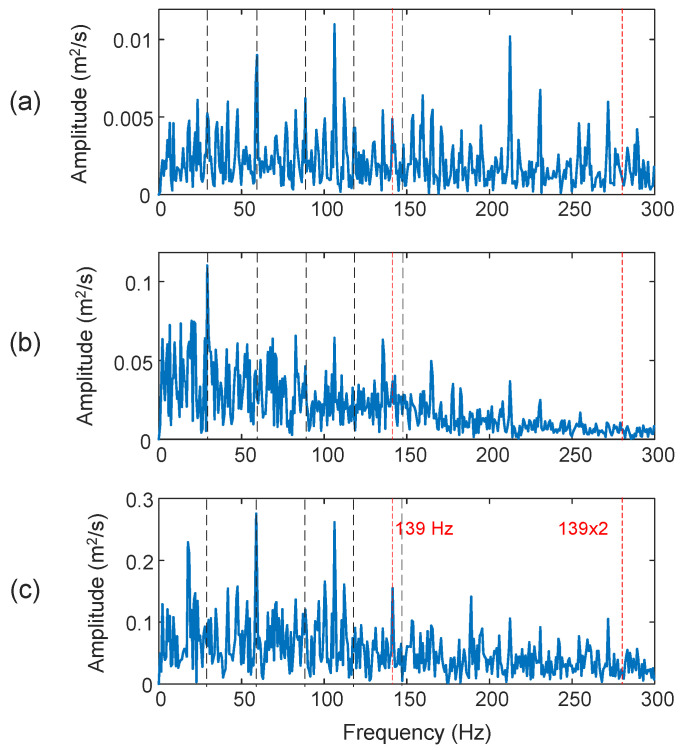
Comparison of processing results of a bearing with a rolling element defect using the techniques of: (**a**) HHT, (**b**) TK, and (**c**) eTK. The red dashed lines represent fault characteristic frequency (139 Hz) and the black dashed lines represent shaft frequency (29.53 Hz) and its harmonics.

Figure 19 shows the processing results using the related techniques for a bearing with a rolling element defect. The characteristic frequency in this case is *f_BD_* = 139 Hz. Both the HHT (*D_I_* = 27.8%) in Figure 19a and the TK method (*D_I_* = 18.6%) in Figure 19b failed to predict the fall fault bearing condition. Although the proposed eTK technique in Figure 19c with denoising filtering can recognize the fault spectral features in this case (*D_I_* = 47.2%), the characteristic frequency component is not a prominent frequency on the spectral map.

## 4. Conclusions

Rolling element bearings are commonly used in rotating machines in both industrial and domestic applications. Reliable bearing fault detection and diagnosis techniques are critically needed in industry to predict bearing faults at their early stages so as to prevent machine performance degradation, improve operation quality, and reduce maintenance costs. A newly enhanced Teager–Kaiser, eTK, technique is proposed in this work for bearing fault detection and diagnosis based on vibration signal analysis. In the proposed eTK technique, (1) an EMD analysis method is suggested to recognize representative IMFs with different frequency components. (2) The eTK denoising filter is proposed to improve the SNR of the selected IMF features. (3) The selected IMFs are synthesized to formulate an analytical signal, which is then applied for signal property analysis and bearing fault detection. (4) The effectiveness of the eTK technique has been verified by experimental tests corresponding to different bearing conditions. (5) Its robustness has been verified by using vibration signals from another experimental setup. Test results show that the eTK denoising filter can improve the SNR of the signal effectively. It can select the most representative IMFs and synthesize them for nonlinear signal property analysis and bearing fault detection. It can also reduce the effects of edge distortion and mode mixing, outperforming other related techniques and showing strong potential tfor real-world machine condition monitoring applications.

## Figures and Tables

**Table 1:** Bearing fault characteristic frequencies in terms of shaft speed $f_{R}$.

Bearing condition	Shaft speed wrt $f_{R}$
Normal/Healthy bearing	$f_{H}=f_{R}$
Outer race fault	$f_{OD}=3.03\times f_{R}$
Inner race fault	$f_{ID}=4.93\times f_{R}$
Rolling element fault	$f_{BD}=3.97\times f_{R}$

**Table 2:** Bearing fault characteristic frequencies in terms of $f_{R}$ in the CWRU setup.

Bearing condition	Shaft speed wrt $f_{R}$
Normal/Healthy bearing	$f_{H}=f_{R}$
Outer race fault	$f_{OD}=3.58\times f_{R}$
Inner race fault	$f_{ID}=5.42\times f_{R}$
Rolling element fault	$f_{BD}=4.71\times f_{R}$

## Data Availability

The data presented in this study are available on request from the corresponding author.
